# Comparative Diagnostic Value of 18F-FDG–PET–CT and Intraoperative Examination in Cervical Cancer Staging

**DOI:** 10.3390/medicina60111758

**Published:** 2024-10-26

**Authors:** Goran Malenković, Jelena Malenković, Sanja Tomić, Armin Šljivo, Fatima Gavrankapetanović-Smailbegović, Slobodan Tomić

**Affiliations:** 1 Faculty of Medicine, University of Novi Sad, 21000 Novi Sad, Serbia; goran.malenkovic@mf.uns.ac.rs (G.M.); jelenam@gmail.com (J.M.); sanja.tomic@mf.uns.ac.rs (S.T.); 2 Clinical Center of University of Sarajevo, 71000 Sarajevo, Bosnia and Herzegovina; sljivo95@windowslive.com (A.Š.); fatima.smailbegovic@gmail.com (F.G.-S.)

**Keywords:** 18F-FDG–PET–CT, cervical cancer, intraoperative exam, staging

## Abstract

*Background and Objectives*: The primary objective of this study is to assess the effectiveness of 18F-FDG–PET–CT in preoperative staging of cervical cancer, focusing on determining surgical operability and exploring the correlation between its quantitative parameters and clinicopathological characteristics. *Materials and Methods*: This retrospective study included 62 cervical cancer patients treated at the Department of Gynecology, Clinic for Operative Oncology at the Institute of Oncology Vojvodina between January 2016 and January 2020, where preoperative clinical examinations and 18F-FDG–PET–CT were performed to assess the extent of cancer, followed by intraoperative and pathohistological examinations of surgically removed specimens to provide a comprehensive evaluation. *Results*: The mean tumor size measured by 18F-FDG–PET–CT was slightly greater than that obtained through clinical examination (26.4 mm vs. 26.0 mm), with a strong linear correlation (r = 0.678, *p* < 0.001) observed between the two measurement methods. The overall prediction accuracy of 18F-FDG–PET–CT for primary tumors is 88.7% (55/62) [sensitivity 86.8%, specificity 100.0%, PPV 100.0%, NPV 56.2%] and for intraoperative examination is 88.7% (55/62) [sensitivity 98.1%, specificity 33.3%, PPV 89.7%, NPV 75.0%]. The agreement with histopathological examination was good for 18F-FDG–PET–CT and moderate for intraoperative examination for primary tumors. Regarding lymph nodes, the overall prediction accuracy of 18F-FDG–PET–CT is 82.2% (51/62) [sensitivity 53.8%, specificity 89.8%, PPV 58.3%, NPV 88.8%] and for intraoperative examination 66.1% (41/62) [sensitivity 76.9%, specificity 63.3%, PPV 35.7%, NPV 91.2%]. The agreement with histopathological examination was moderate for 18F-FDG–PET–CT and poor for intraoperative examination for lymph node metastasis, highlighting that the overall accuracy of 18F-FDG–PET–CT (82.1%) was significantly higher than that of intraoperative examination (66.1%) (*p* = 0.002). *Conclusions*: In conclusion, 18F-FDG–PET–CT provides high accuracy in detecting primary tumors and superior predictive value for lymph node metastases compared to intraoperative examination, highlighting the importance of incorporating this imaging modality into the preoperative evaluation process to enhance diagnostic precision and inform treatment decisions.

## 1. Introduction

Despite the availability of diagnostic procedures for early detection, ongoing advancements in preventive screening programs, and a prolonged pre-invasive period, cervical cancer continues to be a major health concern, ranking as the third most common malignancy among women, with approximately 569,847 new cases and 311,365 deaths each year [1]. According to 2018 data, Serbia registers 1327 new cases of cervical cancer annually, placing it fourth among female malignancies and second among women aged 15 to 44 years [2]. The quest for the most reliable diagnostic algorithm to accurately diagnose, assess the extent of the disease, and develop appropriate therapeutic strategies remains a significant challenge, especially considering that the clinical staging of cervical cancer has historically been based on the 2009 FIGO (International Federation of Gynecology and Obstetrics) classification [3]. However, this system lacked descriptions of distant metastases and did not consider lymph node involvement, which is associated with a poor prognosis [3]. In contrast, the revised 2018 FIGO system emphasized the importance of imaging and permitted its incorporation into the staging process [4,5]. Preoperative imaging techniques offer significant advantages in staging cervical cancer due to their non-invasive nature and accuracy, as they can reliably assess the pre-treatment stage—critical for clinical management and prognosis—demonstrating high sensitivity, specificity, and positive predictive value for metastases of cervical cancer compared to clinical staging [3].

The molecular imaging technique—F18 fluorodeoxyglucose positron emission tomography/computed tomography (18F-FDG–PET–CT) is of great importance in detecting lymph node metastases in cervical cancer [3,4,5]. The functioning principle of this technique is based on the fact that malignant cells increase their glucose utilization rate, resulting in high glycolysis. PET–CT is integral to oncological treatment planning by offering detailed maps of tumor boundaries and metabolic activity. This information enables precise targeting of cancerous tissues during radiation therapy and aids surgeons in planning surgeries to achieve complete tumor resection while minimizing collateral damage to healthy tissue. Additionally, PET–CT serves a critical role in monitoring treatment response post-chemotherapy or radiation [5]. The validity of this method in early-stage patients with resectable tumors has not been sufficiently investigated but is considered the diagnostic method of choice for assessing nodal status in patients with stage II and higher disease and suspected recurrence [5]. The standardized uptake value (SUV), a semi-quantitative measure that provides information about FDG uptake in the examined tissue using PET–CT, can be a parameter for evaluating malignancy and prognosis in cervical cancer such as predicting disease progression, tumor size, extent of the disease, lymph node metastasis, treatment response, and risk of recurrence. Therefore, the application of SUV as a new biomarker, which can be easily measured by PET/CT, has attracted significant attention in the treatment of all gynecological malignancies from the outset. However, the correlation between SUV obtained by PET/CT and histopathological findings in primary tumors has not been significantly studied, and its role in prognosis remains controversial [6,7,8,9]. Several studies have examined SUV as a prognostic factor for uterine cancer patients [10,11]. Numerous studies have evaluated the significance of CT, MRI, and 18F-FDG–PET–CT scans in the initial diagnosis of the extent of cervical cancer and their role in deciding the primary therapeutic modality [12,13,14,15].

The primary objective of our research is to determine the prognostic potential of preoperative 18F-FDG–PET–CT in staging cervical cancer to define its operability. To achieve this, a comparative analysis of preoperative 18F-FDG–PET–CT findings, clinical, intraoperative, and histopathological examinations in detecting lymph node metastases in cervical cancer was conducted.

## 2. Materials and Methods

This retrospective study included patients who underwent surgery at the Department of Gynecology, Clinic for Operative Oncology at the Institute of Oncology Vojvodina in Sremska Kamenica, between January 2016 and January 2020. The data analyzed in this study were collected from available medical records. The sample of patients was formed based on the following criteria: age-appropriate subjects, older than 18 and younger than 65 years, with pathohistological verified invasive cervical cancer assessed by the Oncology Committee for Gynecological Tumors as primarily operable disease stages according to FIGO stages Ia2, Ib1, Ib2, IIa1, and early IIb, who underwent 18F-FDG–PET–CT 7 to 14 days before the surgical treatment for preoperative diagnostic assessment of disease extent. Patients previously treated for malignant diseases, those who received therapy under the cervical cancer treatment protocol before surgical treatment, and those suffering from chronic diseases that contraindicate surgical treatment were excluded from the study.

### 2.1. Methods

The methods used for determining and comparing the extent of cervical cancer included preoperative clinical examination and 18F-FDG–PET–CT, followed by intraoperative examination and pathohistological (PH) examination of the surgically removed specimen.

### 2.2. Clinical Examination

Based on clinical examination parameters, which included speculum examination and bimanual vaginal and rectal examination, the stages considered primarily operable were Ia2, Ib1, Ib2, IIa, and early IIb with initial infiltration of lateral parametria.

### 2.3. F18 Fluorodeoxyglucose Positron Emission Tomography/Computed Tomography (18F-FDG–PET–CT)

The positron emission tomography/computed tomography (PET/CT) examination was conducted using the Biograph 64 True Point system from Siemens Medical Systems, Erlangen, Germany. Subjects received detailed instructions regarding preparation procedures and were intravenously injected with 18F-FDG at a dose adjusted to their body weight (ranging from 185 to 370 MBq or 4.07 MBq × body weight). Following an uptake period of 60–90 min (min–max range), during which patients rested in a specially designed radiation-protected room, they voided their bladders spontaneously before imaging commenced. The imaging protocol began with an initial CT scout scan to determine the scanning range (typically from the skull base to mid-thighs, covering 6–7 positions of the PET acquisition table), followed by a full CT scan for attenuation correction and morphological lesion diagnosis. Subsequently, PET data acquisition was performed using a positron emission detector system. After the acquisition phase, CT and corrected PET tomoscintigrams were reconstructed in axial, sagittal, and coronal projections. Software fusion of CT and corrected PET images was then conducted. The resultant images were analyzed in these three projections alongside the non-corrected PET tomoscintigrams. Special attention was given to tumor size and aggressiveness based on the SUV (standardized uptake value), a semi-quantitative measure of 18F-FDG accumulation used to assess metabolic activity. Lymph node status in pelvic and paraaortic regions and the presence of distant metastatic changes were also carefully evaluated. Regarding the imaging protocol’s reconstruction process, corrections were applied to both CT and PET images for attenuation and scatter corrections, respectively. The images were reconstructed once in a volumetric manner, with corresponding displays in axial, sagittal, and coronal views, facilitating comprehensive analysis. The tumor were delineated by PET–CT scans and the region of interest were drawn manually. When determining the FIGO stage of cervical cancer based on 18F-FDG–PET/CT, lymph node status was integral. Enlarged lymph nodes suspected of malignancy were considered in staging, aligning with the AJCC classification where appropriate (e.g., FIGO stage IIIb).

### 2.4. Intraoperative Examination

For patients with FIGO stages IA2-IIB cervical cancer indicated for surgical treatment, radical hysterectomy (Wertheim-Meigs, Piver type III) was performed. Abdominal opening was conducted via lower medial, paraumbilical, and/or upper medial laparotomy, followed by a detailed inspection and palpation of pelvic and abdominal organs, including lymph nodes in the pelvic and paraaortic regions. The size of the cervix, propagation of cancer to parametria, rectum, and bladder was assessed. Palpable lymph nodes larger than 1 cm in the pelvic and/or paraaortic region were sent for pathohistological analysis. The surgical stage of the disease was determined based on the intraoperative diagnosis, tumor size, parametria involvement, vaginal involvement, and lymph node status.

### 2.5. Pathohistological Examination (PH)

The surgically removed specimen was examined by a pathologist, who reported on the tumor type, size, stromal invasion depth, histological differentiation, lymphovascular invasion, parametrial infiltration, vaginal involvement, number of removed lymph nodes, and number of metastatic lymph nodes in various regions. The AJCC classification was used to stage cervical cancer postoperatively based on the definitive pathohistological findings. The PH parameters were compared with clinical, preoperative 18F-FDG–PET–CT, and intraoperative findings.

### 2.6. Statistical Analysis

Statistical analysis was conducted using SPSS (version 26), employing descriptive methods for qualitative and quantitative assessments, including absolute and relative frequencies, arithmetic mean (X), standard deviation (SD), range, median, and interquartile range (IQR, 25–75%). Depending on data distribution, the statistical significance of differences was assessed using Student’s *t*-test, ANOVA, Mann–Whitney U test, and χ^2^ test, with a significance level set at *p* < 0.05. For test–retest reliability, the paired t-test or Wilcoxon test was utilized. The effectiveness of individual diagnostic methods was evaluated using the Cochrane test, confirming or refuting statistically significant differences. Agreement between diagnostic approaches was assessed using a Kappa statistic and McNemar’s test.

## 3. Results

This study included 62 patients who met the inclusion criteria. The mean age of the patients was 49.3 ± 9.6 years (median 49.0; range 32–75 years).

The clinicopathological characteristics of the participants, summarized in Table 1, show that the most common histological type of cancer was squamous cell carcinoma 59 (95.2%), the most frequent degree of histological differentiation was G2 51 (82.3%), and the predominant clinical stage, according to the FIGO classification, was Ib1 50 (80.6%).

The mean tumor diameter measured by the 18F-FDG–PET–CT (N = 46) was 26.4 mm (SD 10.8; range 10–53 mm), but this could not be established for 16 patients; however, in the remaining 43 patients, the tumor diameter was assessed through both clinical examination and 18F-FDG–PET–CT. Vaginal involvement by 18F-FDG–PET–CT was indicated in only 2 patients (3.2%). The mean value obtained by 18F-FDG–PET–CT was slightly higher than that obtained by clinical examination (26.4 mm vs. 26.9 mm; *p* = 0.784), and there was a good linear correlation between the two methods (r = 0.678, *p* < 0.001). The mean SUV was 10.8 (SD 7.16; range 3.26–45.66) (Table 2).

The 18F-FDG–PET–CT registered increased metabolic activity with suspected metastases in the lymph nodes in the right hemipelvis in 6 (9.7%) patients, with a mean size of 12 mm (SD 6.54; range 8–25 mm) and a mean SUV of 4.35 (SD 1.27; range 2.96–6.59). In the left hemipelvis, increased metabolic activity with suspected metastases in the lymph nodes was registered in 9 (14.5%) patients, with a mean size of 9.67 mm (SD 1.80; range 8–12 mm) and a mean SUV of 3.75 (SD 0.64; range 3.01–4.65). In one patient, 18F-FDG–PET–CT registered increased metabolic activity with suspected metastases in the lymph nodes of the paraaortic region, measuring 6 mm (SUV = 4).

Intraoperative examination revealed suspected tumor involvement of lymph nodes in the right hemipelvis in 22 patients (35.5%), with 18 patients having one suspicious lymph node per location and 4 patients having two (mean of 1.82 lymph nodes per patient). A total of 32 locations of suspicious lymph nodes were registered (obturator—13 (7+3+3); common iliac—12 (4+3+5); external iliac—7 (4+3)). Intraoperative examination for the left hemipelvis indicated suspected tumor involvement of lymph nodes in 22 patients (35.5%), with 18 patients having one suspicious lymph node per location and 4 patients having two (mean of 1.82 lymph nodes per patient). A total of 31 locations of suspicious lymph nodes were registered (obturator—10 (5+3+2); common iliac—12 (4+2+6); external iliac—9 (4+3+2)). Intraoperative examination suspected tumor involvement of lymph nodes in the paraaortic region in 2 patients (3.2%). In 17 patients, there was suspicion that lymph nodes in both hemipelvises (both right and left) were involved, while in 5 patients, suspicion was raised for lymph nodes in either the right or left hemipelvis.

Pathohistological examination confirmed the presence of metastases in the lymph nodes of the right hemipelvis in 9 patients (14.5%), with 6 patients having one metastatic lymph node per location, 1 patient having two, and 2 patients having three (mean of 2 metastatic lymph nodes per patient). A total of 11 locations of metastatic lymph nodes were registered (obturator—3 (1+2); common iliac—6 (1+1+4); external iliac—2 (1+1)). Metastases were confirmed in the lymph nodes of the left hemipelvis in 8 patients (14.5%), with 6 patients having one metastatic lymph node per location, 1 patient having two, and 1 patient having eight (mean of 2 metastatic lymph nodes per patient). A total of 9 locations of metastatic lymph nodes were registered (obturator—4; common iliac—4 (1+3); external iliac—1). Pathohistologically, metastases in the lymph nodes of the paraaortic region were confirmed in one patient (1.6%), with 7 metastatic lymph nodes. Pathohistologically, metastases in the lymph nodes of both hemipelvises (right and left) were confirmed in 4 patients. In 4 and 5 patients, respectively, pathohistologically confirmed metastases were found in the lymph nodes of one side of the hemipelvis—left and right side, respectively (Table 3).

The overall prediction accuracy of the 18F-FDG–PET–CT is 88.7% (55/62) [sensitivity 86.8% (46/53), specificity 100% (9/9), PPV 100% (46/46), NPV 56.2% (9/16)]. (Table 4) False-negative findings occurred in 43.8% (7/16), with no false-positives. The obtained Kappa value of 0.656 (Cohen’s test) indicates good agreement between the 18F-FDG–PET–CT and histopathological examination, while the McNemar test value of *p* = 0.016 indicates asymmetry in the deviations from agreement (Table 5). Regarding intraoperative examination, the overall prediction accuracy is 88.7% (55/62) [sensitivity 98.1% (52/53), specificity 33.3% (3/9), PPV 89.7% (52/58), NPV 75.0% (3/4)]. False-negative findings occurred in 25.0% (1/4), with false-positives in 10.3% (60/58). The agreement between intraoperative examination and histopathological examination of the operative material, based on a Kappa value of 0.409 (Cohen’s test), indicates moderate agreement, while the McNemar test value of *p* = 0.125 suggests nonsignificant asymmetry in deviations from agreement (Table 5).

Cochran’s Q test for tumor presence in three analyses, the 18F-FDG–PET–CT, intraoperative, and histopathological examinations of operative material, yields a value of 18.17 with *p* < 0.001. The overall prediction accuracy of 18F-FDG–PET–CT and intraoperative examination compared to histopathological examination of operative material is the same, at 88.7% (*p* = 1.000). The sensitivity of intraoperative examination is statistically higher than that of 18F-FDG–PET–CT (*p* = 0.014). The specificity of 18F-FDG–PET–CT is statistically higher than that of intraoperative examination (*p* = 0.014) (Table 6).

The overall accuracy of 18F-FDG–PET–CT prediction of lymph nodes is 82.2% (51/62) [sensitivity 53.8% (7/13), specificity 89.8% (44/49), PPV 58.3% (7/12), and NPV 88.8% (44/50)]. False-negative findings occurred in 12.0% (6/50), while false-positives were present in 41.7% (5/12) (Table 7). The Kappa value (Cohen test) of 0.449 indicates moderate agreement between 18F-FDG–PET–CT and the histopathological examination of surgical specimens, and the McNemar test value is *p* = 1.000, suggesting symmetry in deviation from agreement. Six false-negative findings and five false-positive findings were recorded (Table 8).

The overall accuracy of intraoperative examination is 66.1% (41/62) [sensitivity 76.9% (10/13), specificity 63.3% (31/49), PPV 35.7% (10/28), NPV 91.2% (31/34)]. False negative findings occurred in 8.82% (3/34) during intraoperative examination, while false-positives were observed in 64.3% (18/28) (Table 7). The Kappa value (Cohen test) of 0.282 indicates poor agreement between intraoperative examination and the histopathological examination of surgical specimens, and the McNemar test value is *p* = 0.001, suggesting asymmetry in deviation from agreement. Three false-negative findings and 18 false-positive findings were recorded (Table 8).

Cochran’s Q test of the presence of lymph node metastasis in cervical cancer across three analyses—18F-FDG–PET–CT, intraoperative examination, and pathohistological examination of operative material—yields a value of 30.1 with *p* < 0.001, indicating that intraoperative examination significantly raises suspicions of lymph node metastasis in 45.2% of cases compared to 18F-FDG–PET–CT (19.3%) and pathohistological examination of operative material (21.0%). The overall accuracy of 18F-FDG–PET–CT (82.1%) is statistically higher than the overall accuracy of intraoperative examination (66.1%) (*p* = 0.002). The sensitivity of 18F-FDG–PET–CT (53.8%) is not statistically significantly lower than the sensitivity of intraoperative examination (76.9%) (*p* = 0.083). The specificity of 18F-FDG–PET–CT (89.9%) is statistically higher than the specificity of intraoperative examination (63.3%) (*p* < 0.001) (Table 9).

The mean SUV among patients with cervical cancer confirmed by histopathological examination of the operative material was 10.9 (SD = 7.16; min = 3.26; max = 45.6; median = 8.81; 95 IQR = 8.36). Only one participant with histopathological findings of CIS had a measured SUV of 3.72. Therefore, it was not possible to calculate the ROC analysis cut point value for predicting the occurrence of cancer based on the standardized uptake value of the radiopharmaceutical (Table 9). The highest mean SUVs were recorded in participants with squamous cell carcinoma histological type (SUV 11.15), followed by participants with adenocarcinoma histological type (SUV 9.45), and those with adenosarcoma histological type (SUV 8.32). However, the differences observed were not statistically significant (*p* = 0.776; Kruskal–Wallis Test). Regarding the histological differentiation grade, the highest mean SUVs were observed in participants with grade G3 (SUV 11.67), followed by G2 (SUV 11.23), and the lowest in participants with grade G1 (SUV 5.36). While the differences among all three groups were not statistically significant (*p* = 0.095; Kruskal–Wallis Test), there was a statistically significant difference between groups G1 and G2 at a level of *p* = 0.055 (Mann–Whitney U) and between G1 and G3 at a level of *p* = 0.053 (Mann–Whitney U). Regarding the localization in the right/left hemipelvis and the presence or absence of metastases in the lymph nodes, there was no statistically significant difference in the mean SUVs (*p* = 0.592).

## 4. Discussion

The primary treatment for cervical cancer is surgery and radiotherapy, where tumor invasion assessment is critical, and data indicate that in up to 30% of patients, 18F-FDG–PET–CT can alter the initial therapeutic plan [14,15]. Over the last decade, we have witnessed significant advancements in imaging techniques, surgery, and radiotherapy, which, combined with the use of new drugs, have a tremendous impact on the treatment and survival of cervical cancer [16,17]. Following this progress, authors of the revised FIGO staging classification for cervical cancer in 2018 stated that changes were made to reflect routine clinical practice, differentiate prognostic outcomes, and guide treatment stratification. Treatment modalities depend on the stage of the disease and include minimally invasive surgical options that preserve fertility, as well as chemoradiotherapy for locally advanced disease [17,18,19].

The status of pelvic and para-aortic lymph nodes regarding the presence of metastases in cervical cancer is one of the most significant independent prognostic factors in early cervical cancer. Numerous studies have shown that the survival rate of patients with metastases in lymph nodes is lower compared to patients without metastases [13,14,20,21,22,23,24]. The 18F-FDG–PET–CT technique is based on information obtained using a positron emitter labeled as a radiopharmaceutical that distributes throughout the patient’s body. Fluorine-18-fluorodeoxyglucose positron emission tomography with computed tomography is the marker most commonly used in cervical cancer, and we applied it in our study. Regarding the impact of the size of pathological lymph node metastases on survival, a diameter ≥ 20 mm of surgically confirmed metastases in lymph nodes is also associated with a poor prognosis [25]. The nodal ratio, as the percentage of metastatic lymph nodes to total retrieved lymph nodes, is a newly emerged prognostic factor for cervical cancer [26]. Patients participating in our study were eligible for surgical treatment, allowing for postoperative histopathological assessment of lymph nodes.

Processing the collected data within this study yielded a value of 88.7% (55/62) for the overall accuracy of predicting the presence of primary cervical cancer tumors through the 18F-FDG–PET–CT examination compared to the histopathological examination of the operative material. The sensitivity for the presence of primary cervical cancer tumors compared to histopathological examination of the operative material was 86.8% (46/53), while the specificity was 100% (9/9). These findings are supported by data from Choi et al.’s meta-analysis, where the sensitivity and specificity of 18F-FDG–PET–CT in detecting metastases in cervical cancer lymph nodes were 82% and 95%, respectively [6]. In our study, we did not have any false-positive findings, but there were false-negatives. Specifically, the 18F-FDG–PET–CT examination did not detect the primary cervical cancer tumor in 7 cases. The sizes of these tumors ranged from 2 mm to 14 mm. This finding can be explained by the fact that due to reduced spatial resolution, 18F-FDG–PET–CT may give false-negative results for sub-centimeter tumors, as well as surface-infiltrating tumors and micrometastatic secondary deposits that may be detected during MRI examination [27]. A high percentage of false-negative results can be explained by the large percentage of positive lymph nodes smaller than 10 mm in diameter on the histopathological examination of the operative material, as well as the presence of microscopic metastases—small deposits of viable carcinoma cells that are absolutely undetectable by computerized tomography and magnetic resonance imaging. It is known that reactively changed lymph nodes, in terms of hyperplasia or present infection, can also result in false-positive findings [28]. The universally accepted criterion for lymphadenopathy size is a short axis diameter of >10 mm on imaging. However, it has been reported that more than 80% of metastatic lymph nodes are smaller than 10 mm, and more than 50% are smaller than 5 mm [18]. According to these size criteria, lymph node metastases < 10 mm are considered negative, which increases the number of false-negative diagnoses. Sensitivity can be improved by using a threshold of 5 mm, although detecting small lymph nodes can lead to many false-positive cases, resulting in low specificity [29].

Previous studies have shown that 18F-FDG–PET–CT is a hybrid imaging technique, combining functional information from positron emission tomography with anatomical information from computed tomography, which is much more precise and has better sensitivity and specificity compared to computed tomography and/or magnetic resonance imaging scans, especially in detecting the presence of metastases in locoregional lymph nodes and the spread of cervical cancer beyond the pelvis [20,24]. In our study, the maximum specificity value of 100% resulted from the absence of primary cervical cancer tumors during 18F-FDG–PET–CT examination in a total of 9 subjects, as confirmed by histopathological examination of operative material.

After reviewing the data collected during the study, it was found that increased metabolic activity with suspicion of metastases in lymph nodes was registered by 18F-FDG–PET–CT examination in 5 cases of lymph node size 8 mm and SUVs ranging from 2.96 to 4.53. SUV measurements are the most commonly used and generally accepted indices in the published literature for assessing disease activity in various cancers. The results indicate that SUV depends on the histological type of cervical cancer, demonstrating the highest values in squamous cell carcinoma and then in poorly differentiated grade G3 cervical cancer, positively correlating with tumor size but with weak correlation with lymphovascular invasion, which is consistent with previous research [30]. However, many factors can affect the reliability of SUV, such as the time between injection and image acquisition, where two major processes occur: radioactive decay and biological tracer uptake. Usually, after 60 min, FDG uptake is considered mostly constant, and radioactive decay should be accounted for during the analysis. Partial volume effects originate from voxel size; with a given resolution of 5–6 mm of the Biograph, only very few tumors or lymph nodes with a diameter of less than 10–15 mm would be affected at all, yielding lower values which is another reason to look at SUVmax or not use high thresholds for cut-offs, especially in small tumors. Extravasation of injected 18F-FDG at the injection site was not noticed in our images. Residual activity in the syringe was encountered, but we did not provide specific measurements which would affect the results depending on the percentage of residual activity left. Technological characteristics and parameters, such as scanner calibration, image reconstruction algorithms, and acquisition protocols, can also affect the measurements to varying degrees depending on the specific settings used [10,11]. In patients with locally advanced cervical cancer, SUV is an independent prognostic factor that improves discrimination for predicting tumor recurrence and lethal outcomes [31]. Additionally, SUVs of primary tumors could be a prognostic factor for patients undergoing surgery and in early-stage disease. The SUV of PET/CT in patients with primarily large tumors can be used as an independent prognostic biomarker, indicating a reduced rate of survival and disease-free survival period, as well as an aggressive tumor phenotype [32]. The results obtained in our study support the accuracy of the 18F-FDG–PET–CT examination compared to the histopathological examination of the operative material, while the high percentage of intraoperatively determined suspicious lymph nodes is a consequence of the tendency to declare any lymph node larger than 10 mm as suspicious or metastatic. Para-aortic lymphadenectomy was not routinely performed but was indicated only in those cases where increased metabolic activity with suspicion of metastases in lymph nodes was registered by the 18F-FDG–PET–CT examination or if a suspicious lymph node larger than 10 mm was observed during intraoperative examination. A total of two para-aortic lymphadenectomies were performed; one was indicated by the 18F-FDG–PET–CT with increased metabolic activity.

This study had several limitations. Firstly, we utilized a retrospective design, and secondly, the sample size was relatively small. Considering the introduction of new therapeutic treatments, including molecular targeted therapy, further prospective studies are needed.

## 5. Conclusions

Summarizing the results of our study, we can conclude that 18F-FDG–PET–CT showed high specificity in determining the absence of a primary tumor and high complementarity with histopathological findings, implying the potential significance of applying this method in excluding cervical cancer, which needs further verification in a cohort sample in future studies. It has been shown that 18F-FDG–PET–CT has a better overall diagnostic value in detecting metastatic lymph node infiltration compared to intraoperative detection of lymph node metastases based on lymph node size assessment. In our study, 18F-FDG–PET–CT demonstrates statistically significant accuracy in assessing the extent of the underlying disease, based on the results of a statistically significantly lower number of subjects with overestimated disease stage compared to clinical and intraoperative assessment of disease stage, despite the absence of statistically significant differences in the number of subjects with an underestimated disease stage. It is also important to emphasize that 18F-FDG–PET–CT opens the possibility of assessing borderline SUVs to predict the presence of lymph node metastases and consequently identifying patients at increased risk, allowing for modification of treatment modalities and a more personalized therapeutic approach in patients with cervical cancer.

## Figures and Tables

**Table 1:** Clinicopathological characteristics of patients.

	Mean ± SD	Median	Range
Age of onset (years)	49.3 ± 9.6	49.0	32–75
Histological cancer type	N	%	
Squamous cell	59	95.2	
Adenocarcinoma	2	3.2	
Adenosarcoma	1	1.6	
Degree of histological differentiation	N	%	
G1	5	8.1	
G2	51	82.3	
G3	6	9.7	
FIGO	N	%	
Ia2	2	3.2	
Ib1	50	80.6	
Ib2	5	8.1	
IIa1	1	1.6	
IIb	4	6.5	
Total	62	100	

**Table 2:** Mean tumor diameter determined by clinical exam, 18F-FDG–PET–CT, and SUV.

Exam Type	N	Mean ± SD	*p*-Value
Clinical exam	43	26.0 ± 13.2	<0.001
18F-FDG–PET–CT	46	26.4 ± 11.1	
SUV	46	10.8 ± 7.16	

**Table 3:** Distribution of lymph node metastases according to type of examination.

Localization		Exam Type	Localization	
Right hemipelvis		18F-FDG–PET–CT	Left hemipelvis	
N %	N %		N %	N %
Suspect	Normal	Region	Suspect	Normal
5 (8.1)		Common iliac	6 (9.7)	
1 (1.6)		Obturator	3 (4.8)	
6 (9.71)	56 (90.37)	Total	9 (14.5)	53 (85.5)
M (SD)	Min–max		M (SD)	Min–max
1 (0.0)	1–1	Number of suspected lymph nodes	1 (0.0)	1–1
12 (6.54)	8–25	Size of suspected lymph nodes	9.67 (1.8)	8–12
4.35 (1.7)	2.96–6.59	SUV of suspected lymph nodes	3.75 (0.64)	3.01–4.65
N	%	Intraoperative exam	N	%
Suspect		Region	Suspect	
4	18.2	Common iliac–external iliac	4	18.2
3	13.6	Common iliac–obturator	2	9.1
5	22.7	Common iliac	6	27.3
3	13.6	External iliac–obturator	3	13.6
7	31.8	Obturator	2	9.1
22	100	Total	22	100
N	%	Pathohistological exam	N	%
Suspect		Region	Suspect	
1	11.1	Common iliac–external iliac	1	12.5
1	11.1	Common iliac–obturator	0	0
4	44.4	Common iliac	3	37.5
1	11.1	External iliac–obturator	0	0
2	22.2	Obturator	4	50
9	100	Total	8	100

**Table 4:** Comparison of 18F-FDG–PET–CT, intraoperative examination, vs pathological findings regarding the presence of primary tumor of cervical cancer.

Cervical Cancer Primary Tumor Presence							
Modality	SN	SP	PPV	NPV	LPN	LNN	UTP
18F-FDG–PET–CT	86.8% (46/53)	100% (9/9)	100.0% (46/46)	56.2% (9/16)	0.0%	43.8% (7/16)	88.7% (55/62)
Intraoperative exam	98.1% (52/53)	33.3% (3/9)	89.7% (52/58)	75.0% (3/4)	10.3% (60/58)	25.0% (1/4)	88.7% (55/62)

SN—sensitivity; SP—specificity; PPV—positive predictive value; NPV—negative predictive value; LPN—false-positive rate; LNN—false-negative rate; UTP—total accuracy of prediction.

**Table 5:** Presence of primary cervical cancer tumor—comparative analysis.

		**Pathohistological Exam**		
		Cancer Yes	Cancer No	Σ
18 F-FDG-PET–CT	Tu yes	46	0	46
	Tu no	7	9	16
	Σ	53	9	62
		Kappa = 0.656; McNemar Test *p* = 0.016		
		**Pathohistological Exam**		
Intraoperative exam		Cancer yes	Cancer no	Σ
	Tu yes	52	6	58
	Tu no	1	3	4
	Σ	53	9	62
		Kappa = 0.409; McNemar Test *p* = 0.125		

**Table 6:** Cochran’s Q test of the presence of primary cervical cancer tumor.

Variables	N	18F-FDG–PET–CT	OP	PH	Cochran’s Q
Tumor presence	62	46 (74.2%)	58 (93.5%)	53 (85.5%)	*p* < 0.001
Overall accuracy	62	88.7%	88.7%	-	*p* = 1.000
Sensitivity	53	86.8%	98.1%	-	*p* = 0.014
Specificity	9	100%	33.3%	-	*p* = 0.014

**Table 7:** Comparison of 18F-FDG–PET–CT, intraoperative examination, vs pathological findings regarding the presence lymph node metastases of cervical cancer.

Cervical Cancer Primary Tumor Presence							
Modality	SN	SP	PPV	NPV	LPN	LNN	UTP
18F-FDG–PET–CT	53.8% (7/13)	89.8% (44/49)	58.3% (7/12)	88.8% (44/50)	41.7% (5/12)	12.0% (6/50)	82.2% (51/62)
Intraoperative exam	76.9% (10/13)	63.3% (31/49)	35.7% (10/28)	91.2% (31/34)	64.3% (18/28)	8.82% (3/34)	66.1% (41/62)

Numbers represent percentages with a 95% confidence interval. SN—sensitivity; SP—specificity; PPV—positive predictive value; NPV—negative predictive value; LPN—false-positive finding; LNN—false-negative finding; UTP—overall accuracy of prediction.

**Table 8:** Lymph Node Status in Cervical Cancer—Comparative Analysis of 18F-FDG–PET–CT, Pathohistological, and Operative Examination.

		**Pathohistological Exam**		
		Lymph Node +	Lymph Node −	Σ
18 F-FDG-PET–CT	Lymph node +	7	5	12
	Lymph node −	6	44	50
	Σ	13	49	62
		Kappa = 0.449; McNemar Test *p* = 1.000		
		**Pathohistological exam**		
Intraoperative exam		Lymph node +	Lymph node −	Σ
	Lymph node +	10	18	28
	Lymph node −	3	31	34
	Σ	13	49	62
		Kappa = 0.282; McNemar Test *p* = 0.001		

**Table 9:** Cochran’s Q test of the presence of metastases in lymph nodes in cervical cancer.

Variables	N	18F-FDG–PET–CT	OP	PH	Cochran’s Q
PMLN	62	12 (19.3%)	28 (45.2%)	13 (21.0%)	*p* < 0.001
Overall accuracy	62	82.1%	66.1%	-	*p* = 0.002
Sensitivity	13	53.8%	76.9%	-	*p* = 0.083
Specificity	49	89.9%	63.3%	-	*p* < 0.001

PMLN—presence of metastasis in lymph node.

## Data Availability

Data available upon request.

## References

[1] Sung H., Ferlay J., Siegel R.L., Laversanne M., Soerjomataram I., Jemal A. (2021). Global cancer statistics 2020: GLOBOCAN estimates of incidence and mortality worldwide for 36 cancers in 185 countries. CA Cancer J. Clin..

[2] “Institut za Javno Zdravlje Srbije “Dr Milan Jovanovic Batut”. https://www.batut.org.rs/index.php?lang=3.

[3] Pecorelli S., Zigliani L., Odicino F. (2009). Revised FIGO staging for carcinoma of the cervix. Int. J. Gynaecol. Obstet..

[4] Bhatla N., Berek J.S., Cuello Fredes M., Denny L.A., Grenman S., Karunaratne K., Kehoe S.T., Konishi I., Olawaiye A.B., Prat J. (2019). Revised FIGO staging for carcinoma of the cervix uteri. Int. J. Gynaecol. Obstet..

[5] Liu H., Cui Y., Chang C., Zhou Z., Zhang Y., Ma C., Yin Y., Wang R. (2024). Development and validation of a 18F-FDG PET/CT radiomics nomogram for predicting progression free survival in locally advanced cervical cancer: A retrospective multicenter study. BMC Cancer.

[6] Bhatla N., Aoki D., Sharma D.N., Sankaranarayanan R. (2021). Cancer of the cervix uteri: 2021 update. Int. J. Gynaecol. Obstet..

[7] Pujade-Lauraine E., Tan D.S., Leary A., Mirza M.R., Enomoto T., Takyar J., Nunes A.T., Chagüi J.D.H., Paskow M.J., Monk B.J. (2022). Comparison of global treatment guidelines for locally advanced cervical cancer to optimize best care practices: A systematic and scoping review. Gynecol. Oncol..

[8] Cibula D., Pötter R., Planchamp F., Avall-Lundqvist E., Fischerova D., Meder C.H., Köhler C., Landoni F., Lax S., Lindegaard J.C. (2018). The European Society of Gynaecological Oncology/European Society for Radiotherapy and Oncology/European Society of Pathology guidelines for the management of patients with cervical cancer. Int. J. Gynecol. Cancer..

[9] Lin G., Yang L.-Y., Lin Y.-C., Huang Y.-T., Liu F.-Y., Wang C.-C., Lu H.-Y., Chiang H.-J., Chen Y.-R., Wu R.-C. (2019). Prognostic model based on magnetic resonance imaging, whole-tumour apparent diffusion coefficient values and HPV genotyping for stage IB-IV cervical cancer patients following chemoradiotherapy. Eur. Radiol..

[10] Cegla P., Hofheinz F., Burchardt E., Czepczyński R., Kubiak A., Hoff J.V.D., Nikulin P., Bos-Liedke A., Roszak A., Cholewinski W. (2023). Asphericity derived from [18F]FDG PET as a new prognostic parameter in cervical cancer patients. Sci. Rep..

[11] Isaji Y., Tsuyoshi H., Tsujikawa T., Orisaka M., Okazawa H., Yoshida Y. (2023). Prognostic value of 18F-FDG PET in uterine cervical cancer patients with stage IIICr allocated by imaging. Sci. Rep..

[12] Höckel M., Wolf B., Schmidt K., Mende M., Aktas B., Kimmig R., Dornhöfer N., Horn L.-C. (2019). Surgical resection based on ontogenetic cancer field theory for cervical cancer: Mature results from a single-centre, prospective, observational, cohort study. Lancet Oncol..

[13] Hansen H.V., Loft A., Berthelsen A.K., Christensen I.J., Høgdall C., Engelholm S.A. (2015). Survival outcomes in patients with cervical cancer after inclusion of PET/CT in staging procedures. Eur. J. Nucl. Med. Mol. Imaging.

[14] Staley S.A., Tucker K.R., Gehrig P.A., Clark L.H. (2021). Accuracy of preoperative cross-sectional imaging in cervical cancer patients undergoing primary radical surgery. Gynecol. Oncol..

[15] Cibula D., Pötter R., Planchamp F., Avall-Lundqvist E., Fischerova D., Haie-Meder C., Köhler C., Landoni F., Lax S., Lindegaard J.C. (2018). The European Society of Gynaecological Oncology/European Society for Radiotherapy and Oncology/European Society of Pathology guidelines for the management of patients with cervical cancer. Virchows Arch..

[16] Mirpour S., Mhlanga J.C., Logeswaran P., Russo G., Mercier G., Subramaniam R.M. (2013). The role of PET/CT in the management of cervical cancer. Am. J. Roentgenol..

[17] Bhatla N., Singhal S., Dhamija E., Mathur S., Natarajan J., Maheshwari A. (2021). Implications of the revised cervical cancer FIGO staging system. Indian J. Med. Res..

[18] Salib M.Y., Russell J.H.B., Stewart V.R., Sudderuddin S.A., Barwick T.D., Rockall A.G., Bharwani N. (2020). 2018 FIGO Staging Classification for Cervical Cancer: Added Benefits of Imaging. Radiographics.

[19] Manganaro L., Lakhman Y., Bharwani N., Gui B., Gigli S., Vinci V., Rizzo S., Kido A., Cunha T.M., Sala E. (2021). Staging, recurrence and follow-up of uterine cervical cancer using MRI: Updated Guidelines of the European Society of Urogenital Radiology after revised FIGO staging 2018. Eur. Radiol..

[20] Sironi S., Buda A., Picchio M., Perego P., Moreni R., Pellegrino A., Colombo M., Mangioni C., Messa C., Fazio F. (2006). Lymph node metastasis in patients with clinical early-stage cervical cancer: Detection with integrated FDG PET/CT. Radiology.

[21] Kidd E.A., Spencer C.R., Huettner P.C., Siegel B.A., Dehdashti F., Rader J.S., Grigsby P.W. (2009). Cervical cancer histology and tumor differentiation affect 18F-fluorodeoxyglucose uptake. Cancer.

[22] Morice P., Sabourin J.C., Pautier P., Gerbaulet A., Duvillard P., Castaigne D. (2000). Isolated para- aortic node involvement in stage Ib/II cervical carcinoma. Eur. J. Gynaecol. Oncol..

[23] Trimble E. (2009). Cervical cancer state of the clinical science meeting on pretreatment evaluation and prognostic factors, September27-28, 2007:Proceedings and recommendations. Gynecol. Oncol..

[24] Yildirim Y., Sehirali S., Avci M., Yılmaz C., Ertopcu K., Tinar S., Duman Y., Sayhan S. (2008). Integrated PET/CT forthe evaluation of para-aortic nodal metastasis in locally advanced cervical cancer patients with negative conventional CT findings. Gynecol. Oncol..

[25] Kang S., Wu J., Li J., Hou Q., Tang B. (2022). Prognostic significance of clinicopathological factors influencing overall survival and event-free survival of patients with cervical cancer: A systematic review and meta-analysis. Med. Sci. Monit..

[26] Cui H., Huang Y., Wen W., Li X., Xu D., Liu L. (2022). Prognostic value of lymph node ratio in cervical cancer: A meta-analysis. Medicine.

[27] Woo S., Atun R., Ward Z.J., Scott A.M., Hricak H., Vargas H.A. (2020). Diagnostic performance of conventional and advanced imaging modalities for assessing newly diagnosed cervical cancer: Systematic review and metaanalysis. Eur. Radiol..

[28] Liu B., Gao S., Li S. (2017). A comprehensive comparison of, C.T.; MRI, positron emission tomography or positron emission tomography/CT, and diffusion weighted Imaging-MRI for detecting the lymph nodes metastases in patients with cervical cancer: A meta-analysis based on 67 studies. Gynecol. Obstet. Investig..

[29] Benedetti-Panici P., Maneschi F., Scambia G., Greggi S., Cutillo G., D’Andrea G., Rabitti C., Coronetta F., Capelli A., Mancuso S. (1996). Lymphatic spread of cervical cancer: An anatomical and pathological study based on 225 radical hys-terectomies with systematic pelvic and aortic lymphadenectomy. Gynecol. Oncol..

[30] Chong G.O., Jeong S.Y., Lee Y.H., Park S.-H., Lee H.J., Lee S.-W., Hong D.G., Lee Y.S. (2020). Improving the Prognostic Performance of SUVmax in 18F-Fluorodeoxyglucose Positron-Emission Tomography/Computed Tomography Using Tumor-to-Liver and Tumor-to-Blood Standard Uptake Ratio for Locally Advanced Cervical Cancer Treated with Concurrent. J. Clin. Med..

[31] Tan B., Guo J., Wang L., Wang L., Chen B. (2020). Application value of 18F-FDG PETCT imaging in the clinical initial diagnosis and follow-up of primary lesions of cervical cancer. Transl. Cancer Res..

[32] Olthof E.P., Bergink-Voorthuis B.J., Wenzel H.H.B., Mongula J., van der Velden J., Spijkerboer A.M., Adam J.A., Bekkers R.L.M., Beltman J.J., Slangen B.F.M. (2024). Diagnostic accuracy of MRI, CT, and [18F]FDG-PET-CT in detecting lymph node metastases in clinically early-stage cervical cancer—A nationwide Dutch cohort study. Insight Imaging.

